# Comparison of peak oxygen uptake and exercise efficiency between upper-body poling and arm crank ergometry in trained paraplegic and able-bodied participants

**DOI:** 10.1007/s00421-018-3912-1

**Published:** 2018-06-23

**Authors:** Julia Kathrin Baumgart, Laura Gürtler, Gertjan Ettema, Øyvind Sandbakk

**Affiliations:** Centre for Elite Sports Research, Department of Neuromedicine and Movement Science, Norwegian University of Science and Technology, Smistadgrenda 11, 7026 Trondheim, Norway

**Keywords:** Aerobic power, Endurance, Paralympic, Spinal cord injury, VO_2max_, VO_2peak_

## Abstract

**Purpose:**

To compare peak oxygen uptake (VO_2peak_) and exercise efficiency between upper-body poling (UBP) and arm crank ergometry (ACE) in able-bodied (AB) and paraplegic participants (PARA).

**Methods:**

Seven PARA and eleven AB upper-body trained participants performed four 5-min submaximal stages, and an incremental test to exhaustion in UBP and ACE. VO_2peak_ was the highest 30-s average during the incremental test. Metabolic rate (joule/second = watt) at fixed power outputs of 40, 60, and 80 W was estimated using linear regression analysis on the original power-output-metabolic-rate data and used to compare exercise efficiency between exercise modes and groups.

**Results:**

VO_2peak_ did not significantly differ between UBP and ACE (*p* = 0.101), although peak power output was 19% lower in UBP (*p* < 0.001). Metabolic rate at fixed power outputs was 24% higher in UBP compared to ACE (*p* < 0.001), i.e., exercise efficiency was lower in UBP. PARA had 24% lower VO_2peak_ compared to AB (*p* = 0.010), although there were no significant differences in peak power output between PARA and AB (*p* = 0.209).

**Conclusions:**

In upper-body-trained PARA and AB participants, VO_2peak_ did not differ between UBP and ACE, indicating that these two test modes tax the cardiovascular system similarly when the upper body is restricted. As such, the 19% lower peak power output in UBP compared to ACE may be explained by the coinciding lower efficiency.

**Electronic supplementary material:**

The online version of this article (10.1007/s00421-018-3912-1) contains supplementary material, which is available to authorized users.

## Introduction

Peak oxygen uptake (VO_2peak_) and exercise efficiency are key factors for endurance performance. In persons who are primarily able to use their upper-body during exercise, such as many paralympic athletes, the mode most commonly used in assessing VO_2peak_ and efficiency is arm crank ergometry (ACE) (Drory et al. 1990; Glaser et al. 1980; Mossberg et al. 1999; Price et al. 2011; Smith et al. 2001, 2004, 2006, 2007; Tropp et al. 1997). However, sport-specificity of the test mode has been suggested to be of importance for achieving VO_2peak_ and efficiency that are reflective of the endurance capacity in the respective sport (McCafferty and Horvath 1977). For para ice hockey players, sitting para cross-country skiers and para biathletes, the upper-body poling (UBP) movement is the most sport specific. However, it has not yet been investigated whether VO_2peak_ and efficiency differ between ACE and UBP and if possible differences are caused by the respective movement of the arms and/or due to different use of the trunk.

In ACE, power is produced by continuous, asynchronous force application, whereas in UBP—similarly to wheelchair ergometry—power is generated discontinuously, yet in synchronous movements of both hands (Sawka 1986). During ACE, the involvement of the trunk is limited by the asynchronous movement of the hands, whereas during UBP and wheelchair ergometry the synchronous movement of the hands allows more involvement of the trunk. A higher VO_2peak_ may, therefore, be expected in UBP and wheelchair ergometry compared to ACE due to using more muscle mass. However, despite the differences in arm movement and the engagement of the trunk between ACE and wheelchair ergometry, some studies show no differences in VO_2peak_ values between these two modes (Arabi et al. 1997; Gass et al. 1995; Gayle et al. 1990; Glaser et al. 1980; Martel et al. 1991; Price and Campbell 1999b), whereas others report higher values in the wheelchair ergometry mode (Bloemen et al. 2015; Gass and Camp 1984; Sawka et al. 1980). Furthermore, previous studies have shown that wheelchair ergometry is less efficient than ACE (Glaser et al. 1980; Hintzy et al. 2002; Mukherjee and Samanta 2001). This is likely caused by higher coordinative demands of using the discontinuous movement and by production of power during a shorter portion of each cycle in the wheelchair ergometry mode (Mukherjee and Samanta 2001).

Irrespective of the upper-body mode used during exercise testing, VO_2peak_ values were found to be consistently lower in paraplegic (PARA) compared to able-bodied participants (AB) (Price and Campbell 1999a; Hopman et al. 2004; Leicht and Perret 2008). Although the evidence is currently limited, efficiency in both ACE and wheelchair ergometry does not seem to differ between PARA and AB (Glaser et al. 1980).

In the current study, we aimed to compare VO_2peak_ and exercise efficiency between ACE and UBP with the upper-body restricted in both modes in PARA and AB. We hypothesized that VO_2peak_ values would be similar in ACE and UBP, yet lower in PARA as compared to AB. In accordance with the lower efficiency previously found in wheelchair ergometry, exercise efficiency was expected to be lower in UBP compared to ACE in the current study.

## Methods

### Participants

The PARA group consisted of seven (6 men, 1 women) upper-body-trained individuals with a paraplegia and the AB group of eleven (9 men, 2 women) healthy able-bodied upper-body-trained controls (anthropometrics and training hours are presented in Table 1). PARA were significantly older and had significantly lower leg lean muscle mass (LLM) compared to AB (both comparisons, *p* < 0.004). PARA consisted of an ice sledge hockey player, two hand-cyclists, a wheelchair curler, a wheelchair judoist and two recreationally trained participants. AB were sub-elite cross-country skiers who trained 11.5 ± 3.2 h/week, with approximately half of this training spent in modes including the upper-body. Whereas the total number of training sessions was significantly higher in AB (7.2 ± 2.9 sessions/week, *p* = 0.009), training sessions including upper-body exercise did not differ between AB and PARA (4.5 ± 2.4 versus 4.0 ± 1.9 sessions/week, *p* = 0.687). The participants were instructed to refrain from heavy training and alcohol consumption 24 h before the start of the testing, caffeine intake the day of the testing and food intake 2 h before testing. A questionnaire was filled in on each test day to monitor if the participants followed these instructions, as well as to exclude any prior illness or injury that might interfere with the testing. Participants provided written informed consent to voluntarily take part in the study and were informed about the possibility to withdraw from the study at any point in time without providing the reason for doing so. All procedures performed in studies involving human participants were in accordance with the ethical standards of the Regional Ethics Committee for Medical and Health Research in Mid-Norway and with the 1964 Helsinki Declaration and its later amendments. The study was retrospectively registered in the Protocol Registration and Results System (NCT03284086).

**Table 1:** Sex, age, anthropometric and disability characteristics as well as weekly training hours of the participants with paraplegia and the able-bodied participants

	Sex	Age (years)	Height (cm)	Body mass (kg)	Lean muscle mass (kg)			Disability (level)	ASIA score	Training (h/week)
					Arms (left + right)	Trunk	Legs (left + right)			
1	Male	26	177	73.5	8.7	32.2	12.5	Paraplegia (L2)	C	5.0
2	Male	27	178	59.4	7.2	26.2	13.7	Paraplegia (Th10)	D	6.0
3	Male	19	165	79.2	9.5	31.8	7.7	Spina bifida (N/A)	N/A	7.0
4	Male	48	195	95.0	10.6	37.6	14.1	Paraplegia (Th9)	A	8.0
5	Female	40	168	65.0	5.1	21.2	12.0	Paraplegia (Th3)	D	6.0
6	Male	43	178	83.5	9.7	32.6	11.5	Paraplegia (L1)	A	1.8
7	Male	28	192	76.4	8.2	34.1	13.1	Paraplegia (L1)	A	3.5
Mean ± SD		33.8 ± 11.2	179 ± 11	74.4 ± 12.5	8.4 ± 1.8	30.8 ± 5.4	12.1 ± 2.1			5.7 ± 2.0
1	Male	26	183	79.1	9.1	33.7	23.4			14
2	Female	21	171	72.3	5.7	26.8	18.9			16
3	Female	20	168	56.3	4.8	22.5	13.5			11
4	Male	22	178	72.2	6.9	31.5	18.7			12.5
5	Male	24	183	75.5	7.2	30.7	20.9			6
6	Male	22	187	78.1	7.7	34.5	22.2			15
7	Male	26	184	80.4	9.4	34.7	24.2			10
8	Male	21	190	92	9.6	36.4	25.5			12.5
9	Male	19	180	70	7.6	29.6	20.1			14
10	Male	23	183	76.3	7.7	32.8	22.8			8
11	Male	19	184	79.6	8.1	33.1	23.6			ns
Mean ± SD		22.4 ± 2.6	183 ± 3	78.1 ± 6.2	8.1 ± 1.0	33.0 ± 2.1	22.4 ± 2.2			11.5 ± 3.2

American Spinal Cord Injury Association (ASIA), not applicable (N/A), not specified (ns)

### Overall design

The testing consisted of two test days, where participants performed four 5-min submaximal steady-state stages, an incremental test to exhaustion and a verification stage in UBP or ACE in a counterbalanced order. Tests were performed at the same time of day to minimize the bias of diurnal variation in performance (Atkinson and Reilly 1996). The time between tests was a minimum of 48 h and a maximum of 4 days. On a separate day before or after the testing, body composition was assessed using dual-energy X-ray absorptiometry (DXA).

### Test set-up

**Fig. 1:**
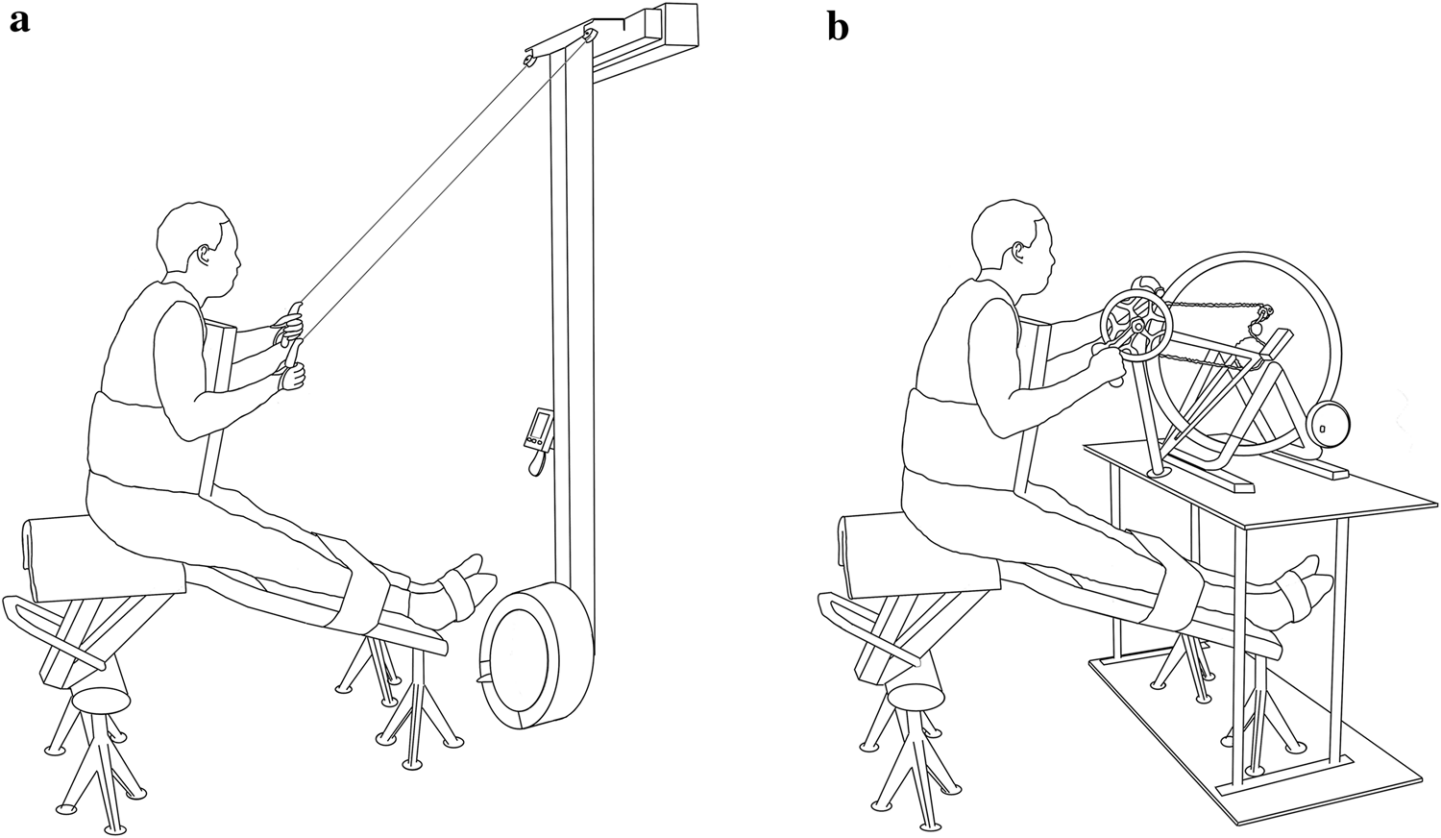
Test set-up with the participant in a sitting position with the upper-body and the legs fixed in front of the Concept2 ski-ergometer (**a**) and the arm crank ergometer (**b**)

After being equipped with an oro-nasal mask (Hans Rudolph Inc, Kansas City, MO, USA) and a heart rate monitor (Polar Electro Inc., Port Washington, NY, USA), participants were tightly strapped into a seat construction, which consisted of a modified weight lifting bench placed in front of the UBP or ACE ergometer (see Fig. 1a, b). The upper-body was fixed during all tests to limit differences in involvement of the trunk between UBP and ACE as well as AB and PARA. Furthermore, the legs were supported and fixed to minimize leg muscle activation. The spiroergometer (Oxycon Pro, Jaeger, Viasys BV, Bilthoven, the Netherlands) was calibrated against a known mixture of gases (15% O_2_, 5% CO_2_). The flow transducer was calibrated with a 3-L syringe (Calibration syringe D, SensorMedics, Yorba Linda, CA, USA). Respiratory parameters were assessed by open-circuit calorimetry, with expired gases passing through a mixing chamber and being measured continuously. The Concept2 ski-ergometer (Concept2, Morrisville, USA) was used during testing in the UBP mode. An ErgStick (Endurance Sports Research Limited, United Kingdom) was connected to the PM4 monitor of the Concept2 ski ergometer and the application Float (ErgStick Ltd, United Kingdom) continuously recorded power output (PO) and stroke rate. The ergometer’s software has previously been validated with force and velocity measurements (Hegge et al. 2015). The ACE was custom-made from a road-bike (White, XXL Sport & Villmark AS, Norway) and equipped with an electronical brake system for indoor cycling (CompuTrainer™, RacerMate®, Inc., Seattle, USA). The crank axis was aligned with the participant’s shoulder height and the seat positioned so that the participant’s elbows were slightly bent at maximal reach. The tire pressure was kept stable at six bars and the CompuTrainer™ was calibrated prior to each test session. The in-built software (PerfPRO Studio©, Dynastream Innovations Inc., Canada) continuously recorded PO and crank rate.

### Test protocol

#### Submaximal stages

Prior to testing, participants familiarized themselves with the test set-up by 5 min of arm cranking or upper-body poling at low intensity [overall rating of perceived exertion (RPE) 8–9]. The testing then commenced by performing four times 5-min submaximal stages at overall RPEs of 9 (very light), 11 (light), 13 (somewhat hard) and 15 (hard) on a 6–20 Borg scale (Borg 1982). Target RPE at increasing intensities from 9 to 15 (Hegge et al. 2015) was used instead of fixed workloads to ensure that the participants covered a similar range of exercise intensities relative to their maximal capacity. In the ACE mode, crank rate was self-chosen within 60–90 revolutions per minute, whereas stroke rate in the UBP mode was fully self-chosen.

Oxygen uptake (VO_2_), respiratory exchange ratio (RER) and minute ventilation (VE) were recorded as 10-s averages. Heart rate (HR) was recorded every second. PO was recorded every second in ACE, and for every stroke in UBP and then interpolated at 1-s intervals. After each submaximal stage, there was a 2- to 3-min break during which a 20 µL blood sample was taken from the fingertip and blood lactate (BLa) measured with the Biosen C-Line Sport lactate measurement system (EKF-diagnostic GmbH, Magdeburg, Germany). Furthermore, overall RPE was recorded. Steady-state PO, VO_2_, RER, VE, and HR, were calculated by averaging the values during the last 2 min of each submaximal stage. There are three primary ways to describe mechanical efficiency during exercise: delta efficiency, net efficiency and gross efficiency. In brief, the challenges with net efficiency and delta efficiency, which are outlined more in detail by Ettema and Lorås (2009), concern the assumption that the processes related to the resting metabolism are independent of the processes associated with producing work. In comparison, gross efficiency, which is the ratio of PO and metabolic rate (MR), is a theoretically sound concept. However, it is affected by the diminishing effect of the resting metabolism with increasing PO. Therefore, we also consider the entire PO-MR relationship to interpret exercise efficiency in the current study.

MR in joule/second (watt) was calculated from VO_2_ and RER with the use of a standard conversion table (Peronnet and Massicotte 1991). MR was then estimated from the original PO-MR relationship at a PO of 40, 60 and 80 W using linear regression analyses in Matlab R2016a (MathWorks Inc., Natic, USA). The resulting MR outcomes were used to investigate exercise efficiency between exercise modes and groups. In addition, gross efficiency was calculated as MR divided by PO at 40, 60 and 80 W.

#### Incremental test to exhaustion

After a 5-min passive break and a 3-min active recovery at the workload equivalent to the first stage (RPE 9), the participants performed an incremental test to exhaustion. The incremental test started at the individual PO of the second submaximal stage (RPE 11) (rounded to the nearest 10-W value) of the respective mode. PO was then increased by 10 W every 1 min. Termination criteria were a drop in PO and a plateau (3 values with < 2 mL·kg^− 1^·min^− 1^ difference) (Taylor et al. 1955) or drop in VO_2_ (> 2 mL·kg^− 1^·min^− 1^). BLa was measured 1 and 3 min after the incremental test. Furthermore, overall RPE was recorded directly after the incremental test. After a 5-min passive break and a 3-min active recovery, participants performed a verification stage where they directly increased the workload to the peak PO of the incremental test to verify that no higher VO_2peak_ can be obtained despite a longer duration spent at high workload (Leicht et al. 2013).

30-s moving averages were calculated for the PO and the respiratory parameters and the highest values were defined as peak values. 3-s moving averages were calculated for the HR data and the highest value defined as peak HR. The higher of the two blood lactate values was defined as peak BLa.

### Statistics

A linear mixed model with fixed coefficients and random intercept was employed to investigate the effect of exercise mode, group and exercise intensity on PO, physiological and perceptual parameters during the submaximal stages and the incremental test. This model investigates the effect of one factor (exercise mode or group or exercise intensity) while adjusting for the two other factors. The same model was used to investigate the effect of exercise mode and group on exercise efficiency. Paired-samples *T* tests were used to compare gross efficiency between exercise modes and groups at each of the three POs. An alpha level of 0.05 was used to indicate statistical significance. IBM SPSS Statistics 24.0 (SPSS Inc., Chicago, USA) was used for all statistical analyses.

## Results

### Peak values from incremental test

An overview of the peak values reached during the incremental test is provided in Table 2. During UBP, when group was adjusted for, participants produced 19% lower peak PO compared to ACE (*p* < 0.001) but displayed 0.08 higher RER (*p* < 0.001). PARA had a 24% lower VO_2peak_ (*p* = 0.010) and 1.2 higher RPE (*p* = 0.018) compared to AB. However, peak PO did not significantly differ between PARA and AB despite being 14% lower in PARA compared to AB (*p* = 0.209). A significant interaction in peak VE existed between exercise mode and group (*p* = 0.049). When investigating each group separately, AB displayed a trend towards a 22% higher peak VE in UBP compared to ACE (*p* = 0.069), whereas in PARA there was no significant difference between modes (*p* = 0.804).

**Table 2:** Peak power output, peak physiological and perceptual responses (Mean ± SD) during the incremental test to exhaustion in the upper-body poling and arm crank ergometry mode in the seven paraplegic and eleven able-bodied participants

	Upper-body poling		Arm crank ergometry	
	Paraplegic	Able bodied	Paraplegic	Able bodied
Peak PO (Watt)	118 ± 34	118 ± 34	146 ± 33*	146 ± 33*
	104 ± 35	127 ± 31	136 ± 38	152 ± 29
VO_2peak_ (mL·kg^− 1^·min^− 1^)	35.9 ± 7.8	35.9 ± 7.8	37.3 ± 8.0	37.3 ± 8.0
	30.3 ± 6.1	39.5 ± 6.6^†^	32.7 ± 7.0	40.3 ± 7.3^†^
Peak VE (L·min^− 1^)	145 ± 37*	145 ± 37*	126 ± 40	126 ± 40
	131 ± 47	154 ± 28	124 ± 44	126 ± 39
Peak RER	1.19 ± 0.05*	1.19 ± 0.05*	1.11 ± 0.06	1.11 ± 0.06
	1.20 ± 0.05	1.19 ± 0.06	1.15 ± 0.06^†^	1.09 ± 0.04
Peak HR (beats·min^− 1^)	176 ± 16	176 ± 16	178 ± 17	178 ± 17
	172 ± 20	178 ± 14	182 ± 14	176 ± 18
Peak BLa (mmol·L^− 1^)	10.8 ± 1.9	10.8 ± 1.9	9.9 ± 3.0	9.9 ± 3.0
	10.2 ± 2.3	11.3 ± 1.5	10.3 ± 3.7	9.6 ± 2.6
RPE (6–20)	18.8 ± 1.2	18.8 ± 1.2	18.4 ± 1.2	18.4 ± 1.2
	19.3 ± 0.5	18.5 ± 1.4	19.2 ± 0.6^†^	17.8 ± 1.2

Power output (PO), peak oxygen uptake (VO_2peak_), minute ventilation (VE), respiratory exchange ratio (RER), heart rate (HR), blood lactate (BLa), overall rating of perceived exertion (RPE)

*Significantly higher in either upper-body poling or arm crank ergometry at an alpha level of 0.05

^†^Significantly higher in either able-bodied or paraplegic participants at an alpha level of 0.05

### Submaximal values

**Fig. 2:**
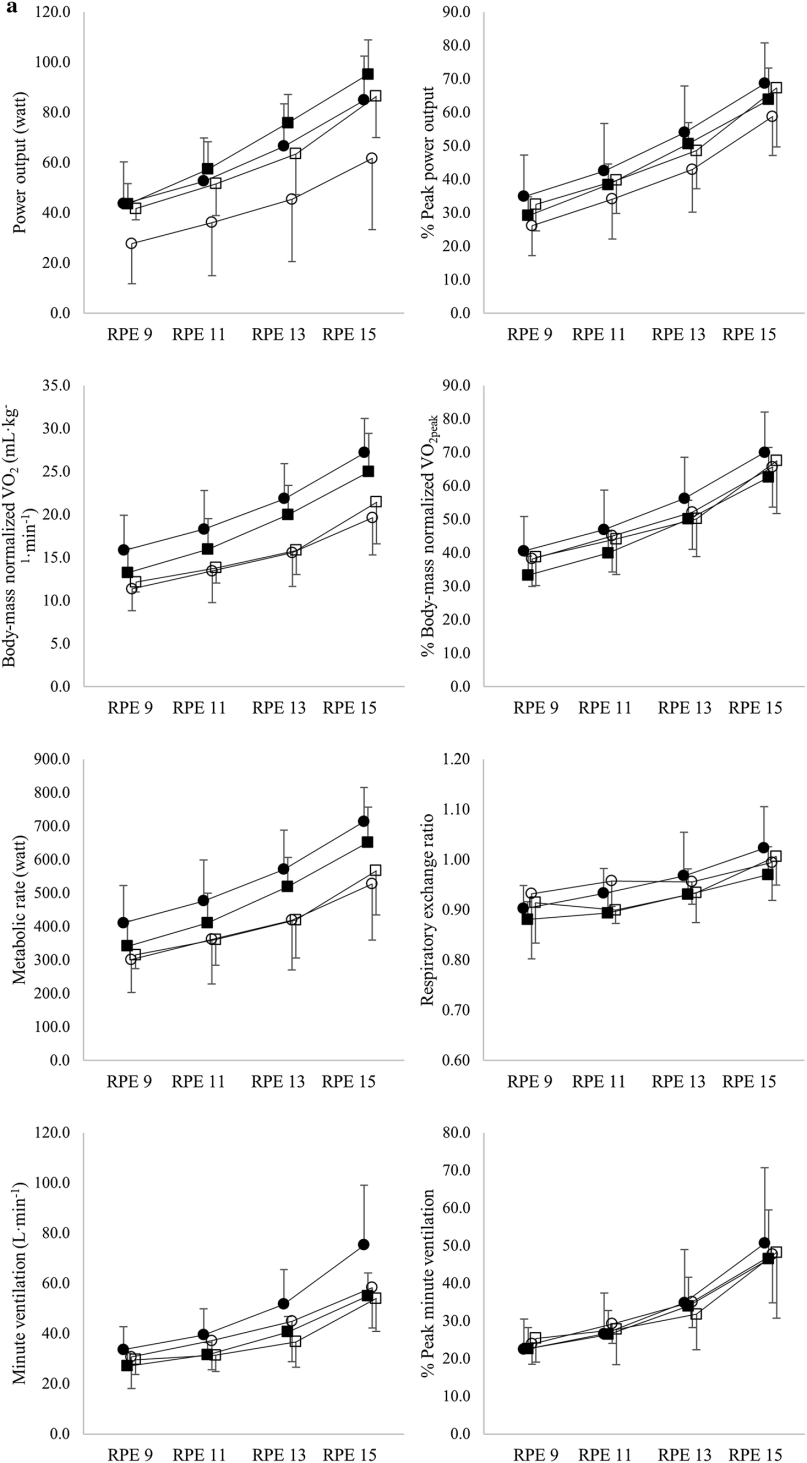

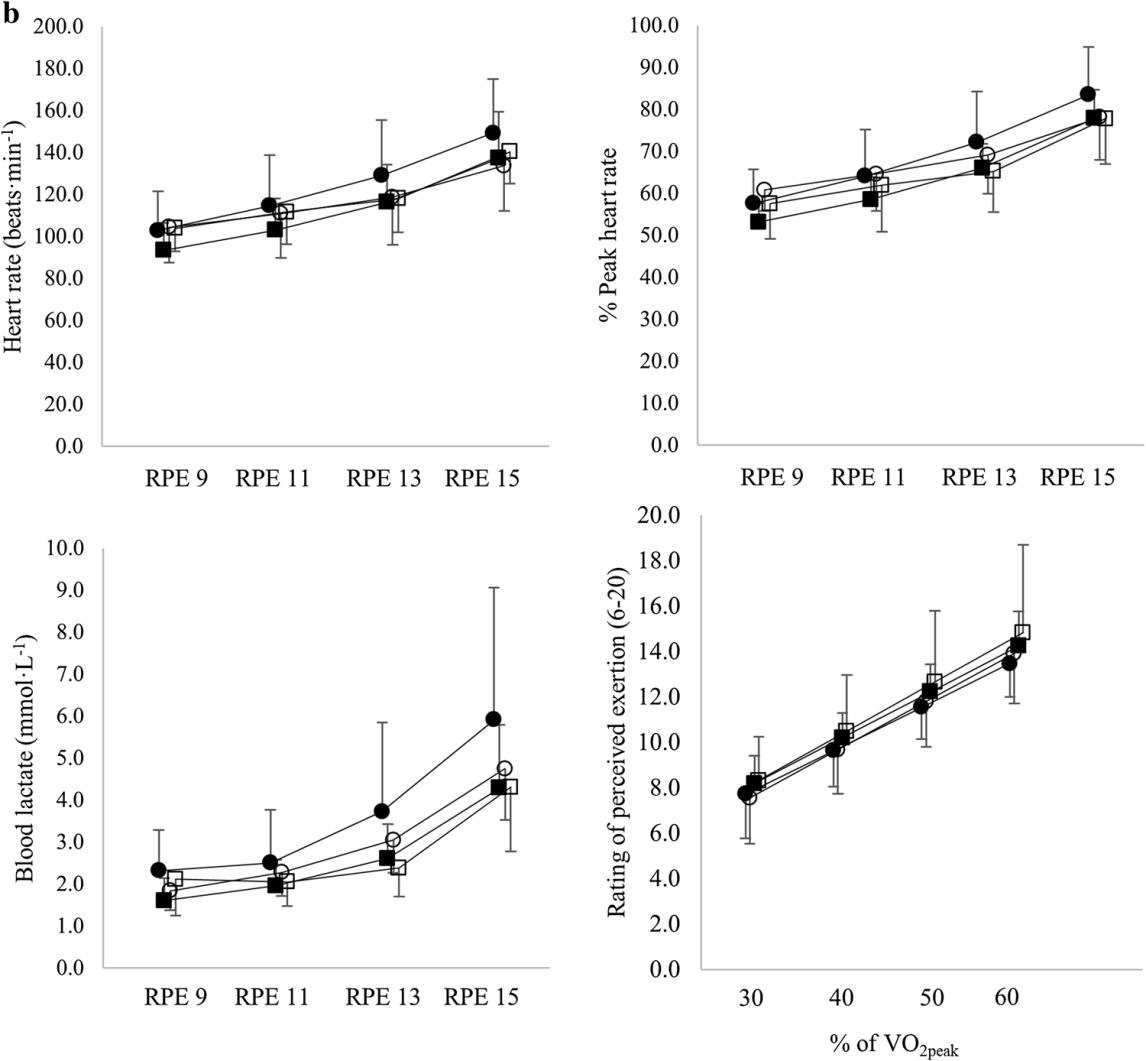
Power output and physiological parameters at a rating of perceived exertion (RPE) of 9, 11, 13 and 15 presented both as absolute values and as percentage of peak. Furthermore, RPE is presented at 30, 40, 50 and 60% of peak oxyen uptake (VO_2peak_) (Circles represent the UBP mode, squares the ACE mode. Open symbols represent the PARA participants, closed symbols the AB participants). Oxygen uptake (VO_2_)

All outcome parameters significantly increased from the first stage (RPE 9) to the fourth stage (RPE 15) (all comparisons, *p* < 0.001) (Fig. 2a, b). During UBP, at a given RPE, participants produced 16% lower PO (*p* < 0.001) and displayed 7% higher VO_2_ (*p* = 0.005), 9% point higher % of VO_2peak_ (*p* < 0.001), 8% higher MR (*p* = 0.001), 0.04 higher RER (*p* < 0.001), 19% higher VE (*p* < 0.001), 6% higher HR (*p* = 0.001), 7% point higher % of peak HR (*p* < 0.001) and 0.50 mmol·L^− 1^ higher BLa (*p* = 0.002) compared to ACE. PARA had a trend towards 18% lower PO (*p* = 0.081) and displayed 20% lower VO_2_ (*p* = 0.016) and 22% lower MR (*p* = 0.046). No significant differences between neither modes nor groups were found in % of peak PO and % of peak VE (all comparisons, *p* > 0.689). No significant differences between groups were found in % of VO_2peak_, VE, RER, HR, % of peak HR and BLa (all comparisons, *p* < 0.283). Furthermore, no significant differences in RPE at 30, 40, 50 and 60% of VO_2peak_ were found (*p* = 0.993).

### Exercise efficiency

**Fig. 3:**
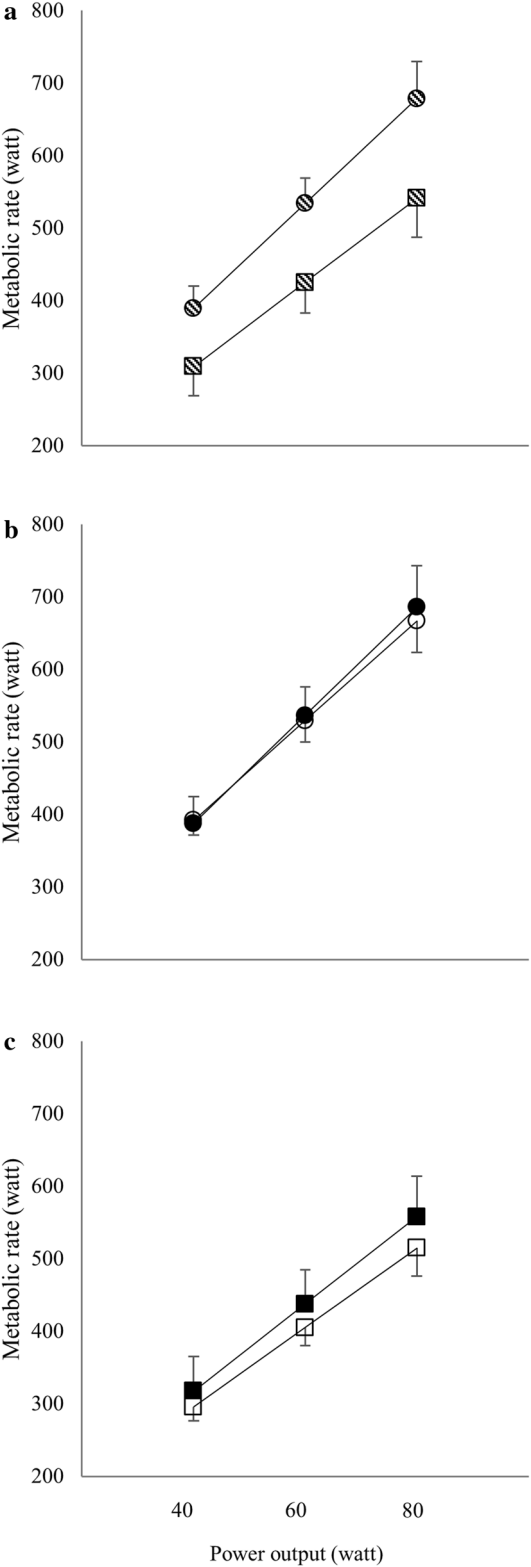
Metabolic rate–work rate relationship in the comparisons of **a** upper-body poling (circles) and arm crank ergometry (squares) with paraplegic and able-bodied participants pooled, **b** paraplegic (open circles) and able-bodied participants (closed circles) in the upper-body poling mode, and **c** paraplegic (open squares) and able-bodied participants (closed squares) in the arm crank ergometry mode

MR was 24% higher in UBP compared to ACE (*p* < 0.001), i.e., exercise efficiency was lower in UBP (Fig. 3a). In line with this, gross efficiency calculated at 40, 60 and 80 W was significantly lower in UBP (10.4 ± 0.9, 11.4 ± 0.8 and 12.0 ± 0.9) compared to ACE (12.9 ± 1.8, 14.0 ± 1.8 and 14.7 ± 1.9) (all comparisons *p* < 0.001). MR was not significantly different between PARA and AB (*p* = 0.323) (Fig. 3b, c).

## Discussion

The aim of this study was to compare VO_2peak_ and exercise efficiency between upper-body poling (UBP) and arm crank ergometry (ACE) in paraplegic (PARA) and able-bodied (AB) participants. As expected, VO_2peak_ did not differ between UBP and ACE, indicating that both modes tax the cardiovascular system similarly. However, there was a 19% lower peak power output (PO) produced in UBP that coincided with the 24% higher metabolic rate (MR) at a given PO (i.e., lower gross efficiency). PARA did not differ from AB in exercise efficiency, but PARA had 24% lower VO_2peak_ compared to AB.

### Differences between UBP and ACE

This is the first study to investigate differences in VO_2peak_ and exercise efficiency between UBP and ACE. We found no difference in VO_2peak_ between UBP and ACE, which indicates that—with the upper-body restricted—the cardiorespiratory system is taxed equally in both exercise modes when working until voluntary exhaustion. In addition, no differences in peak HR, peak BLa and RPE were found between UBP and ACE, indicating that a similar level of exhaustion was reached at the end of the tests. However, although the peak aerobic energy delivery capacity and the ability to reach exhaustion did not differ between exercise modes, peak PO was clearly lower in UBP compared to ACE. The difference in peak PO is likely explained by UBP being a less efficient test mode, which is also supported by lower efficiency in UBP compared to ACE at submaximal workloads.

The higher MR at a given power (i.e., lower gross efficiency) in UBP may be related to that power is produced in a discontinuous movement, which includes larger fluctuations in instantaneous power, compared to in ACE where the movement is more continuous. In line with this, studies comparing wheelchair propulsion to ACE have found that the discontinuous movement during wheelchair propulsion is less efficient (Hintzy et al. 2002; Glaser et al. 1980; Mukherjee and Samanta 2001). Discontinuous force application has been shown to increase power fluctuations within strokes, a strategy that costs more for the production of the same PO (Glaser et al. 1980). Furthermore, in UBP, the participants move their arms up against gravity before pulling down on the ropes. This movement fundamentally differs from ACE where the arms are supported by the cranks throughout the whole cycle, a movement pattern that has previously been associated with higher exercise efficiency compared to wheelchair propulsion (Mukherjee and Samanta 2001), arguably due to reutilization of kinetic energy. Altogether, the lower exercise efficiency in UBP compared to ACE may be explained by the different movement characteristics.

The % of peak PO employed at the various RPE-matched submaximal stages was almost identical between UBP and ACE. However, UBP showed a higher MR and a trend towards lower absolute PO during each of these stages, which is associated with the lower exercise efficiency in UBP. In addition, all related physiological outcome parameters (e.g., VO_2_, MR, RER, VE, HR, BLa) were significantly higher at a given RPE during UBP compared to ACE. Therefore, the greater physiological stress during UBP might be related to differences in local metabolic responses in the working upper-body muscles, such as a higher local oxygen desaturation and a lower local muscle blood flow (this is indicated by unpublished data from our research group), in response to the higher instantaneous power production during each stroke in the UBP compared to the ACE mode. However, further studies measuring local muscle blood flow and desaturation are needed to investigate this hypothesis.

### Differences between AB and PARA

As expected, PARA displayed significantly lower VO_2peak_ compared to AB, which might partially be due to a more limited ability to recruit muscle mass during testing. Even though we tried to minimize differences in trunk and leg stabilization between PARA and AB, we still observed leg muscle contractions in AB especially towards the end of the incremental test. In addition, VO_2peak_ might be lower in PARA due the inability to redistribute blood from the paralyzed trunk and lower limbs, which is related to a reduced stroke volume and, at maximal exercise intensities, to a reduced cardiac output (Hopman et al. 1992, 1993; Thijssen et al. 2009). In PARA with an injury level above Th6, VO_2peak_ may be further restricted due to reduced blood redistribution from the splanchnic vascular bed (Thijssen et al. 2009). Furthermore, the lower VO_2peak_ in PARA may be related to the fact that AB perform more overall training hours. Whereas the amount of upper-body training did not differ between PARA and AB and the two groups had a similar amount of muscle mass in the upper body, AB had twice the amount of overall training hours due to additional exercise with lower-body- and whole-body exercise modes. Additionally, PARA consisted of a group of athletes from different sports, whereas AB were all cross-country skiers. As such, PARA might be less specifically trained for the upper-body poling movement, and one might expect the difference in VO_2peak_ to be bigger in UBP as compared to ACE. However, differences in VO_2peak_ were similar between PARA and AB both in UBP and ACE. This indicates that, when the upper-body is restricted, sports-specificity does not seem to have a major effect on VO_2peak_.

There was no difference in MR at a given PO between PARA and AB, indicating that PARA were equally efficient as AB. In addition, AB had a higher VO_2_ but also a comparably higher absolute PO at all RPE-matched submaximal stages. Hence, as a % of VO_2peak_ and of peak PO, participants exercised at the same relative intensity in both groups. Furthermore, none of the other physiological parameters significantly differed between PARA and AB when expressed as a percentage of peak values. Concluding from the above, differences in the submaximal responses between AB compared to PARA are due to AB working at higher PO and not due to differences in exercise efficiency.

### Methodological considerations

While the fixed position of the upper body reduced potential differences in the use of the muscles of the trunk and pelvic region between UBP and ACE as well as AB and PARA, it likely influenced VO_2peak_ and other related outcome parameters as well. Not restricting upper-body movement (as is more commonly seen when UBP is used during training and competition), would have led to a different use of the trunk in UBP compared to ACE, in particular in the comparison of PARA versus AB. In UBP, trunk movement can easily contribute to increased power production and thereby elevated MR (Hegge et al. 2015). In comparison, due to the asynchronous arm movements in ACE, there is a lower contribution of the trunk movement to power production. Further studies are needed to compare the effect of fixed trunk versus allowing the trunk to move freely on VO_2peak_ during UBP incremental exercise to exhaustion.

## Conclusion

In upper-body trained PARA and AB participants, VO_2peak_ did not differ between UBP and ACE, indicating that the movement patterns of these two test modes tax the cardiovascular system to a similar extent when the trunk is fixed. The 19% lower peak PO in UBP compared to ACE may be explained by the coinciding lower efficiency in UBP. Furthermore, the lower VO_2peak_ in PARA compared to AB is likely related to their disability, i.e. less active muscle mass during testing and a limited blood redistribution below the level of injury. However, there was no difference in exercise efficiency between PARA and AB in the two modes. Overall, the findings of this study provide a good starting point for understanding the differences in outcome parameters between two commonly used test modes and between PARA and AB athletes. However, to allow coaches and researchers to implement our findings into practice, future research should complement our results by investigating whether differences in trunk involvement between UBP and ACE lead to differences in VO_2peak_ and efficiency.

## Electronic supplementary material

Below is the link to the electronic supplementary material.

Supplementary material 1 (XLSX 159 KB)

## Abbreviations

AB: Able-bodied participants
ACE: Arm crank ergometry
BLa: Blood lactate
HR: Heart rate
MR: Metabolic rate
PARA: Participants with a paraplegia
PO: Power output
RER: Respiratory exchange ratio
RPE: Overall rating of perceived exertion
UBP: Upper-body poling
VE: Minute ventilation
VO_2_: Oxygen uptake
VO_2peak_: Peak oxygen uptake
